# Eluent Tolerance and Enantioseparation Recovery of Chiral Packing Materials Based on Chitosan Bis(Phenylcarbamate)-(*n*-Octyl Urea)s for High Performance Liquid Chromatography †

**DOI:** 10.3390/molecules21111528

**Published:** 2016-11-13

**Authors:** Jing Wang, Shao-Hua Huang, Wei Chen, Zheng-Wu Bai

**Affiliations:** 1 School of Chemistry and Environmental Engineering, Wuhan Institute of Technology, Wuhan 430073, China; wjdj9288@163.com (J.W.); wchen@wit.edu.cn (W.C.); 2 Key Laboratory of Biobased Materials, Qingdao Institute of Bioenergy and Bioprocess Technology, Chinese Academy of Sciences, Qingdao 266101, China; huangsh@qibebt.ac.cn

**Keywords:** chitosan, chiral packing material, durability, chiral recognition, high performance liquid chromatography

## Abstract

The goal of the present work was to study the influence of the swelling of chitosan derivatives on the enantioseparation and the separation performance recovery of chiral stationary phases (CSPs) based on these derivatives. Therefore, six chitosan bis(phenylcarbamate)-(*n*-octyl urea)s were synthesized, which were coated on macroporous 3-aminopropyl silica gel affording new CSPs. Most of the CSPs demonstrated strong enantioseparation capability for the tested chiral compounds. The swelling capacity of the chitosan bis(phenylcarbamate)-(*n*-octyl urea)s in ethyl acetate, acetone and tetrahydrofuran (THF) was evaluated. Among the chitosan derivatives, the chitosan bis(3,5-dichlorophenylcarbamate)-(*n*-octyl urea) polymer showed the highest swelling capacity in ethyl acetate and THF. The polymer-based CSPs could be utilized with pure ethyl acetate and a normal phase containing 70% THF, but was damaged by pure THF. On the other hand, the separation performance of the damaged CSP could be recovered after it was allowed to stand for a period of time. The observations are important for the development and application of polysaccharide derivative-based CSPs.

## 1. Introduction

Enantioseparation plays a very important role in fundamental research in the chemical and pharmaceutical industry [1,2,3,4,5]. A well-developed technique to achieve enantioseparation for detection and preparation purposes is high-performance liquid chromatography assisted by chiral packing materials, i.e., chiral stationary phases (CSPs). CSPs are usually prepared from a solid support (mostly silica gel), on which a chiral selector (CS) is coated or covalently immobilized. Enantioseparation is thus implemented by the chiral recognition between the chiral selector and chiral compound enantiomers. Among commercialized CSPs, those made from the derivatives of cellulose and amylose have attracted much more attention, due to their strong chiral recognition capability and wide applicability [6,7,8,9]. To date, lots of works on the preparation, chiral recognition mechanism and application of the CSPs derived from cellulose and amylose have been reported [10,11,12,13], and on those of the CSPs made with cellulose tris(3,5-dimethylphenylcarbamate) (CDMPC) and amylose tris(3,5-dimethylphenylcarbamate) (ADMPC) in particular [14,15,16,17]. To overcome the drawback of dissolution or high swelling of chiral selectors in chromatographic mobile phases, cellulose/amylose derivatives are covalently immobilized on a support [18,19,20]. The generated CSPs, however, sometimes display relatively lower chiral recognition capability, depending on the cross-linker employed and the degree of cross-linking. Therefore, chiral packing materials are preferably prepared by coating rather than covalent immobilization. To achieve this goal, polysaccharide derivatives must be insoluble in common organic solvents, and not to be highly swollen in these solvents. From the reported literature, chitin/chitosan derivatives may meet these requirements. Okamoto and coworkers have synthesized chitin bis(arylcarbamate)s with various aryl isocyanates [21], chitosan bis(arylcarbamate)-(aryl urea)s [22], and chitosan bis(arylcarbamate)-(phthaloyl imide)s [23]. Most of these chitin/chitosan derivatives barely dissolved in many organic solvents that could be used as mobile phase components. Recently, we reported chitosan bis(arylcarbamate)-(alkyl amide)s [24,25], chitosan bis(arylcarbamate)-(alkoxy formamide)s [26,27] and chitosan bis(3,5-dimethylphenylcarbamate)-(alkyl urea)s [28,29]. The as-synthesized chitosan derivatives hardly dissolved in most organic solvents depending on the molecular weight of the chitosans employed as the starting material. Moreover, many of these chitosan derivatives demonstrated good enantioseparation capability. In particular, the chitosan bis(3,5-dimethylphenylcarbamate)-(*n*-octyl urea) CSP displayed both satisfactory enantioseparation capability and high tolerance to “unusual mobile phases” [29].

In practice, the polymers of polysaccharide derivatives swell in organic solvents more or less even if they cannot be dissolved. If the corresponding CSPs are analyzed with a mobile phase in which the chiral selectors swell, whether the chiral recognition capability of the CSPs can recover or not is unknown, particularly for the CSPs prepared from polysaccharide derivatives which highly swell in mobile phases. This is not only an interesting issue, but also a topic that requires urgent clarification, because it relates to CSPs design and mobile phase choice for enantioseparation. To probe this issue, in the present work, a series of chitosan bis(arylcarbamate)-(*n*-octyl urea)s were synthesized, from which the related new CSPs were prepared. Their swelling capacity and enantioseparation capability were then evaluated. It was found that chitosan bis(3,5-dichlorophenylcarbamate)-(*n*-octyl urea) exhibited the highest swelling capacity, while the relevant CSP provided very good enantioseparation for the tested chiral compounds. The recovery in enantioseparation capability of this CSP after being exposed to “unusual mobile phases” was also preliminarily investigated and discussed.

## 2. Results and Discussion

### 2.1. Characterization of Chitosan Derivatives

**Scheme 1:**
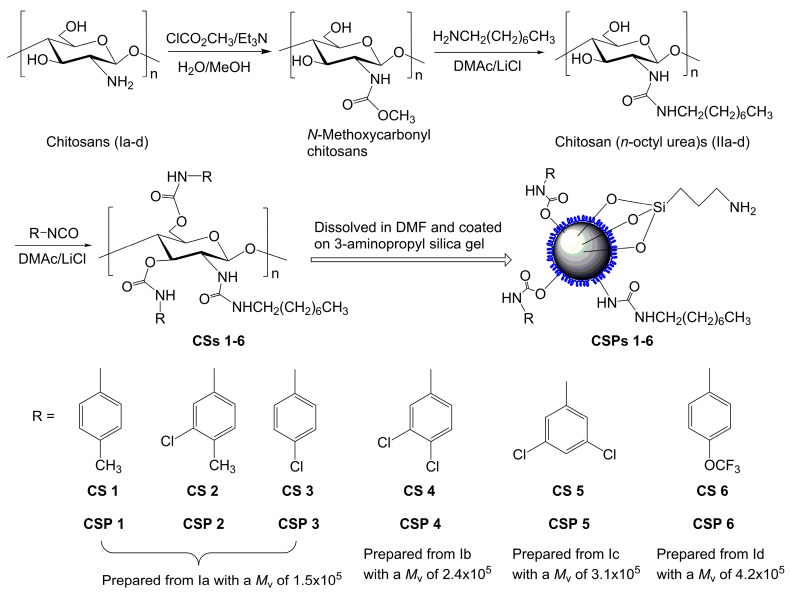
Preparation scheme for chitosan bis(arylcarbamate)-(*n*-octyl urea)s and corresponding CSPs. The different colors means the silica gel (the ball) and the chitosan derivatives coated on the silica gel.

**Figure 1:**
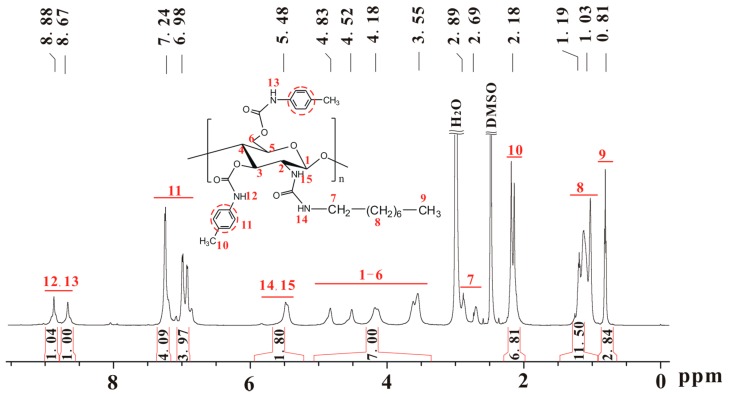
^1^H-NMR spectrum of chitosan bis(4-methylphenylcarbamate)-(*n*-octyl urea) (**CS 1**).

Scheme 1 shows the synthetic scheme and structures of the prepared chitosan derivatives. The chitosans with different molecular weights used as the starting material were prepared by the deacetylation of chitin in aqueous and pentanolic NaOH solution. The typical ^1^H-NMR spectra, represented by Figure S1 (see Supplementary Materials) show that the chitosans were almost completely deacetylated. The essential intermediates, i.e., chitosan (*n*-octyl urea)s (**IIa**–**d**), were characterized in a previous study and the degree of substitution of *n*-octyl group was found to be nearly 100% [28]. Figure 1 shows the ^1^H-NMR spectrum of chitosan bis(4-methylphenylcarbamate)-(*n*-octyl urea) (**CS 1**), in which the integrals match the proton numbers. The same observations can be found for the ^1^H-NMR spectra of **CSs 2**–**6** (Figures S2–S6). Additionally, the calculated values for elemental analysis of the chitosan bis(phenylcarbamate)-(*n*-octyl urea)s are close to those of the found ones. The IR spectra of **CSs 1**–**6** are presented in Figure S7, in which some typical absorbance bands can be found. The above characterizations evidence that **CSs 1**–**6** were obtained as expected.

### 2.2. Swelling Capacity of the Chiral Selectors

Conventional coated-type CSPs of cellulose/amylose derivatives (e.g., CDMPC and ADMPC) can only work in a limited range of mobile phases, because the derivatives dissolve or highly swell in some usual organic solvents, such as ethyl acetate, acetone and THF, etc. Although the CSPs possess very strong enantioseparation capability, they cannot be used with mobile phases containing the above-mentioned solvents. Therefore, for polysaccharide-derivative based CSPs, the dissolution and swelling of the polysaccharide derivatives in common solvents must be seriously considered. In most cases, the derivatives of cellulose and amylose are easily dissolved in THF. Many coated-type CSPs were accordingly prepared with THF as the solvent to dissolve the derivatives [21,22,23,30]. On the contrary, THF in a mobile phase will undoubtedly damage the CSPs. From the reported literatures, we can conclude THF damages coated-type CSPs more than other solvents, except for dimethyl sulfoxide, DMAc and DMF. The latter three highly polar solvents are seldom employed as mobile phase components for usual HPLC analysis. If a polysaccharide type CSP can work in THF-containing mobile phases, it almost also surely be utilized with mobile phases containing other usual solvents.

**Figure 2:**
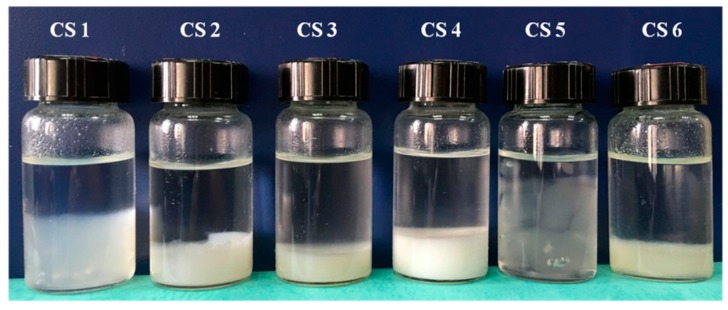
Swelling states of **CSs 1**–**6** in THF.

The swelling capacity of **CSs 1**–**6** is exhibited in Table 1, in which ethyl acetate, acetone and THF were selected as the test solvents. As shown in Table 1, **CSs 1**–**6** demonstrated lower swelling capacity in ethyl acetate and acetone than in THF. Figure 2 shows the morphologies of **CSs 1**–**6** swollen in THF. From Table 1 and Figure 2, **CS 5**, i.e., chitosan bis(3,5-dichlorophenylcarbamate)-(*n*-octyl urea), swelled the most in THF. Based on the swelling capacity of **CSs 1**–**6** in these solvents, **CSs 1**–**6** might be used with mobile phases containing a high proportion of ethyl acetate and acetone or even with these two pure solvents, and might be used with mobile phases containing a certain amount of THF [29].

### 2.3. Enantioseparation Capability of ***CSPs 1**–**6***

**Figure 3:**
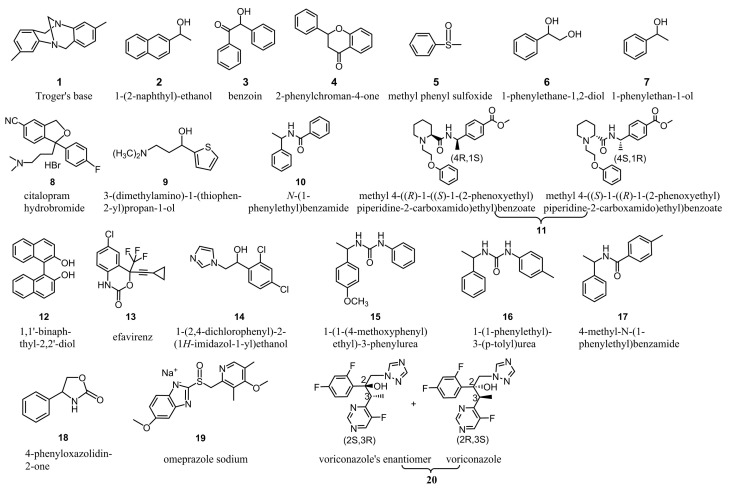
Structures of chiral analytes tested.

**Figure 4:**
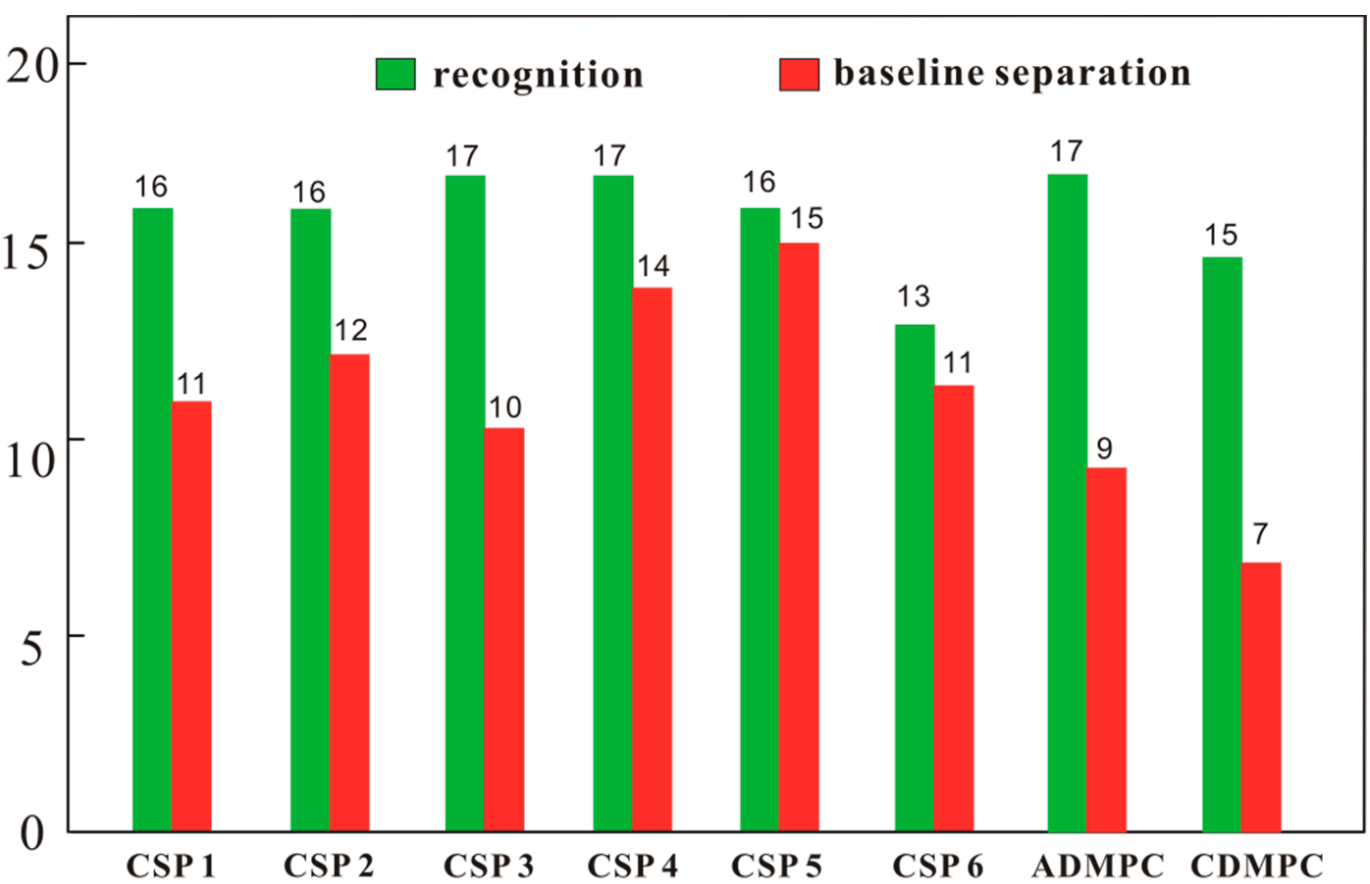
Total numbers of chiral analytes recognized and baseline separated by **CSPs 1**–**6**, ADMPC and CDMPC in the mobile phases of *n*-hexane/isopropanol (90/10), *n*-hexane/ethanol (90/10) and *n*-hexane/ethanol/methanol (90/5/5). Column size: Φ 250 mm × 4.6 mm; flow rate: 1 mL/min; detection temperature: 25 °C.

After **CSPs 1**–**6** were packed into empty columns, the column efficiencies were tested with 1,1′-biphenyl as the solute in *n*-hexane/isopropanol (90/10). The column efficiencies were in the range of 22,400–40,000 plates per meter, which were satisfactory for chromatographic analysis. The enantioseparation capability of **CSPs 1**–**6** was evaluated with twenty chiral compounds (Figure 3) and three mobile phases including *n*-hexane/isopropanol (90/10), *n*-hexane/ethanol (90/10) and *n*-hexane/ethanol/methanol (90/5/5). Based on the numbers of chiral compounds recognized (α > 1.0) and baseline separated (R_s_ ≥ 1.50), the enantioseparation capabilities of **CSPs 1**–**6** can be judged and compared. The chromatographic separation results of **CSPs 1**–**6**, ADMPC and CDMPC are presented in Tables S1 and S2, and the numbers of chiral compounds recognized and baseline separated are counted and displayed in Figure 4. As shown in Figure 4, **CSPs 1**–**5** all demonstrated good enantioseparation capability compared to ADMPC and CDMPC for the tested chiral samples, whereas **CSP 6** exhibited relatively lower enantioseparation capability. **CSP 4** chirally recognized 17 chiral compounds (85% of the total) and baseline separated 14 of them (70% of the total); **CS 5** recognized 16 chiral compounds (80% of the total), and baseline separated 15 of them (75% of the total). These two CSPs thus showed preferable enantioseparation capability.

### 2.4. Eluents Tolerance and Enantioseparation Recovery of ***CS 5***

Among the prepared chiral selectors, **CS 5**, i.e., chitosan bis(3,5-dichlorophenylcarbamate)-(*n*-octyl urea) showed highest swelling capacity in THF. In ethyl acetate and acetone, the swelling capacities of the **CSs 1*–*6** were not remarkably different. Due to these facts, the tolerance of the related CSP (**CSP 5**) against organic solvents should be the poorest. Therefore, **CSP 5** was selected for the investigation of its eluent tolerance according to the previously reported method [24,25]. After the completion of enantioseparation evaluation in the mobile phases of *n*-hexane/isopropanol (90/10), *n*-hexane/ethanol (90/10) and *n*-hexane/ethanol/methanol (90/5/5), the column packed with **CSP 5** was allowed to stand for one month, and the condition of the corresponding **CSP 5** was named “I”. Then the enantioseparation capability of the **CSP 5** under condition I was evaluated again in the three mobile phases (Table S3). As can be seen in Tables S1 and S3, the chromatographic results of **CSP 5** obtained in the second analysis are generally close to the ones obtained in the first time. Afterwards, **CSP 5** was flushed with pure ethyl acetate at a flow rate of 1 mL/min for 7 h, and the condition of **CSP 5** exposed to this treatment was called “II”; the condition of **CSP 5** then flushed with *n*-hexane/THF (30/70) was named “III”, and called “IV” after **CSP 5** was finally flushed with pure THF. Under the conditions II, III, and IV, the enantioseparation of **CSP 5** was tested with the twenty chiral compounds using in *n*-hexane/ethanol (90/10). The enantioseparation results of **CSP 5** obtained under conditions I–IV with *n*-hexane/ethanol (90/10) as the mobile phase are displayed in Table 2.

**Figure 5:**
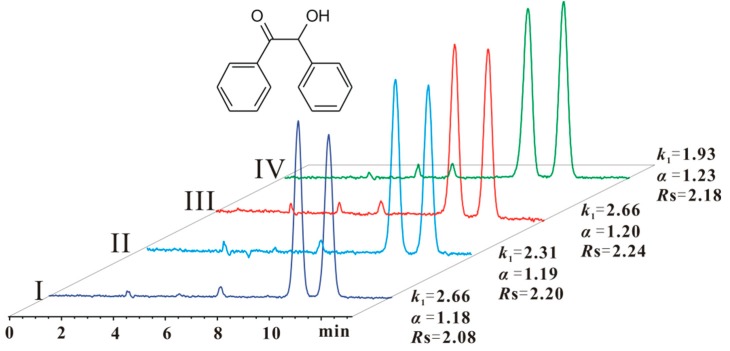
Enantioseparation of compound **3** (benzoin) on **CSP 5** under different conditions. The conditions of **CSP 5**: I: after **CSP 5** had been analyzed with *n*-hexane/isopropanol (90/10), *n*-hexane/ethanol (90/10) and *n*-hexane/methanol/ethanol (90/5/5), and then was allowed to stand for one month; II: after **CSP 5** had been analyzed in condition I and then flushed with pure ethyl acetate; III: after **CSP 5** had been analyzed in condition II and then flushed with *n*-hexane/THF (30/70); IV: after **CSP 5** had been analyzed in condition III and then flushed with pure THF. Eluent: *n*-hexane/ethanol (90/10). Column size: Φ 250 mm × 4.6 mm; flow rate: 1 mL/min; detection temperature: 25 °C, UV wavelength: 254 nm.

As shown in Table 2, after **CSP 5** was flushed with pure ethyl acetate or *n*-hexane/THF (30/70), no significant difference in the resolution (*R*_s_) was observed for most of the chiral compounds. The resolution of some chiral compounds (e.g., compounds **4** and **11**) was reduced a little, whereas that of some compounds (e.g., compounds **3** and **14**) increased. However, after **CSP 5** was flushed with pure THF, the resolution of most chiral compounds decreased to a different extent (Table 2 (IV)). For example, compounds **1** and **17** were no longer baseline separated. On the other hand, there were still two exceptional examples: compound **3** was separated with close resolution under all conditions (Figure 5); compound **11** was not baseline separated after **CSP 5** was flushed with ethyl acetate and *n*-hexane/THF (30/70), but was baseline separated after flushing with pure THF (Table 2 (II, III and IV)). In summary, **CSP 5** could tolerate pure ethyl acetate and 70% THF mobile phases, but was damaged by pure THF. Anyway, the maximum content of THF in a normal phase can reach 70% for the enantioseparation by **CSP 5**. Grinberg et al. investigated the suprastructure variation of ADMPC and CDMPC under different conditions, and found that the suprastructures varied in different solvents [31]. Therefore, we presume that the suprastructure of **CS 5** on **CSP 5** should be altered in different mobile phases. This variation might impact the complexation between chiral analytes and **CS 5** leading to the difference in enantioseparation. This impact should be relevant to the structure of chiral compounds, which was concluded on the basis of the above-mentioned enantioseparation characteristics of compounds **3** and **11** under different conditions.

**Figure 6:**
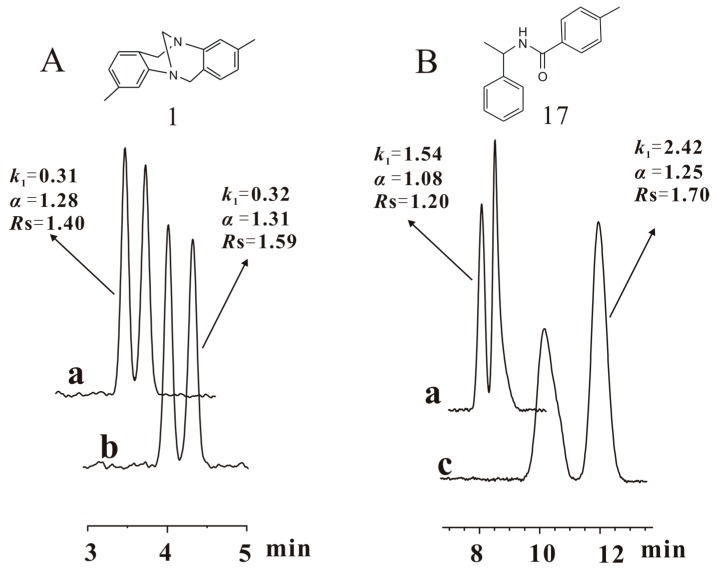
Chromatograms of compounds **1** (Tröger´s base) (**A**) and **17** (**B**) baseline separated again by **CSP 5** under condition V with changed mobile phases. a: obtained in the mobile phase (*n*-hexane/ethanol (90/10)) for general enantioseparation evaluation under condition V; b: obtained in the specifically changed mobile phase (*n*-hexane/ethanol (93/7)) for baseline separation of compound **1** under condition V; c: obtained in the specifically changed mobile phase (*n*-hexane/ethanol/isopropanol (90/5/5)) for baseline separation of compound **17** under condition V. Column size: Φ 250 mm × 4.6 mm; flow rate: 1 mL/min; detection temperature: 25 °C, UV wavelength: 242 nm.

After **CSP 5** was flushed with pure THF and then tested for its enantioseparation, the chiral column was filled with *n*-hexane/isopropanol (90/10) and allowed to stand for two months (condition V). In order to fully investigate the enantioseparation capability under condition V, **CSP 5** was analyzed again with *n*-hexane/isopropanol (90/10), *n*-hexane/ethanol (90/10) and *n*-hexane/ethanol/methanol (90/5/5) (Table S3). Comparing the separation results obtained in conditions IV and V with *n*-hexane/ethanol (90/10) as the mobile phase (Table 2), it was observed that the resolution of most chiral compounds increased and recovered to the level achieved in condition III. Besides, the separation results achieved in conditions I and V, respectively, with *n*-hexane/isopropanol (90/10), *n*-hexane/ethanol (90/10) and *n*-hexane/ethanol/methanol (90/5/5) as the mobile phases are presented in Table S3. Comparing the resolutions available in the three mobile phases, the enantioseparation performance of **CSP 5** under condition V was on the whole equivalent to that in condition I. The above observations mean that the separation power of **CSP 5** was restored during the standing period. Compounds **1** and **17** were not baseline separated again in *n*-hexane/ethanol (90/10) although **CSP 5** was allowed to stand for two months after it was damaged by pure THF. An attempt to baseline separate these two compounds again was made by changing the alcohol or alcohol ratio in the mobile phase. When the ethanol content reduced from 10% to 7%, compound **1** was baseline separated again; the baseline separation of compound **17** was also achieved in the mobile phase of *n*-hexane/ethanol/isopropanol (90/5/5), in which 5% ethanol was replaced by 5% isopropanol (Figure 6). This fact reveals that the optimal mobile phase for the enantioseparation of a few chiral analytes might be altered a little after **CSP 5** was flushed with pure THF and then put aside for two months.

## 3. Experimental Section

### 3.1. Materials and Instruments

Macroporous silica gel (particle size: 7 μm, pore size: 1000 Å) was purchased from Daiso Co., Ltd (Osaka, Japan). All substituted phenyl isocyanates, including 4-methylphenyl, 3-chloro-4-methyl-phenyl, 4-chlorophenyl, 3,4-dichlorophenyl, 3,5-dichlorophenyl and 4-trifluoromethoxyphenyl isocyanate were purchased from Puyang Hongda Shengdao New Materials Co., Ltd (Puyang, China). Chitin (from shrimp shell) was commercially obtained from Hai Zhi Yuan Biological Products Co., Ltd (Weifang, China). Prior to being deacetylated, chitin was washed with a diluted hydrochloric acid solution and water in sequence and finally was dried. The dried chitin was smashed into powder [25]. The chitosans with different molecular weights were prepared by changing the reaction condition during deacetylation of chitin [32,33]. The viscosity-average molecular weights (*M*_v_) of the prepared chitosans were estimated with an Ubbelohde viscometer with 0.1 M aqueous acetic acid solution as the solvent. *N*,*N*-Dimethylacetamide (DMAc), 4-*N*,*N*-dimethylaminopyridine (DMAP), triethylamine, toluene, *N*,*N*-dimethylformamide (DMF), lithium chloride and molecular sieve (4 Å) were analytic grade. These reagents as well as the organic solvents used for chromatographic separation were purchased from Sinopharm Chemical Reagent Co., Ltd (Shanghai, China). The DMAc and toluene were dried over molecular sieve three times. The lithium chloride was dehydrated in vacuum at 140 °C for 24 h. 3-Aminopropyltriethoxysilane was purchased from J&K Scientific Ltd. (Beijing, China). 3-Aminopropyl silica gel was prepared by the reaction of the macroporous silica gel and the 3-aminopropyltriethoxysilane in the dried toluene [34]. Chiral analytes were kindly provided by Huahai Pharmaceuticals Co., Ltd (Linhai, China), Nantong General Pharmaceutical Co., Ltd (Nantong, China) and Nhwa Pharmaceutical Co., Ltd (Xuzhou, China), or were purchased from J&K Scientific Ltd., or were synthesized according to a previously reported method [35]. Deuterated trifluoroacetic acid (TFA-*d*) (D, 99.5%) and dimethyl sulfoxide (DMSO-*d*_6_) (D, 99.5%) used for ^1^H-NMR spectroscopy measurements were purchased from Cambridge Isotope Laboratories Inc. (Andover, MA, USA).

The ^1^H-NMR spectrum of chitosan was recorded on a 400 MHz NMR spectrometer from Varian (Palo Alto, CA, USA). The sample solutions (about 12 mg in 0.5 mL) were prepared with TFA-*d* as the solvent, which was also employed as the reference for proton (δ 11.50 ppm). Detection temperature was set at 25 °C. ^1^H-NMR spectra of chitosan bis(arylcarbamate)-(*n*-octyl urea)s were measured on an Avance III 600 MHz high-resolution liquid NMR spectrometer (Bruker, Karlsruhe, Germany) with a 5 mm TCI CryoProbe equipped with Z-gradients up to 53 G/cm. The sample solutions (7 mg in 0.5 mL) were prepared with DMSO-*d*_6_ as the solvent in the presence of tetramethylsilane. All IR spectra were measured on a PerkinElmer infrared spectrometer (PerkinElmer, Waltham, MA, USA) as KBr pellets. The contents of carbon, hydrogen and nitrogen of chitosan derivatives were detected on an Elemental VarioEL III CHNOS analyzer (Elementar Analysensysteme, GmbH, Germany). Empty stainless steel columns (Φ 250 mm × 4.6 mm) for HPLC were commercially available from Thermo Fisher Scientific Inc. (Waltham, MA, USA). The as-prepared CSPs were packed into the columns with an Alltech Model 1666 slurry packer (Alltech, Chicago, IL, USA). The enantioseparation capability of the CSPs was evaluated on an Agilent 1100 chromatographic apparatus (Agilent, Santa Clara, CA, USA), which was equipped with an Agilent G1365B DAD, an Agilent G1311A quaternary pump, an Agilent G1379A degasser, and an Agilent G1313A ALS autosampler.

### 3.2. Preparation and Characterization of Chitosan Derivatives

*N*-Methoxycarbonyl chitosans and chitosan (*n*-octyl urea)s were synthesized according to the method described in a previous work [28]. A series of chiral selectors (CSs) based on the chitosan (*n*-octyl urea)s were synthesized by the following general procedures with various structurally different isocyanates. Briefly, a chitosan (*n*-octyl urea) was dissolved in a solution of LiCl in DMAc (10% by weight) at 80 °C, to which four equivalents of an isocyanate (2 mol isocyanate to 1 mol hydroxyl group) was added. The resulting solution was stirred at 85 °C for 30 h. The crude product was obtained as the insoluble fraction of the reaction solution in methanol, and purified by reprecipitation with DMF to dissolve the crude product and methanol as the precipitating agent. The reprecipitation was conducted at least three times till the filtrates was free from organic substance that was detected by thin-layer chromatography. Finally, the product was washed with water and then dried in vacuum.

*Chitosan bis(4-methylphenylcarbamate)-(n-octyl urea)* (**CS 1**), yield: 92%; IR (KBr, cm^−1^) υ: 3400, 3325 (–CONH–), 2954, 2854 (–C–H), 1721 (–CO_2_–), 1599 (–Ph), 1657, 1527 (–CONH–); ^1^H-NMR (δ, ppm, 600 MHz, DMSO-*d*_6_, 90 °C): 8.88, 8.67 (2H, –NH–CO_2_–), 7.34–6.82 (8H, Ph–H), 5.48 (2H, –NH–CO–NH–), 5.07–3.36 (7H, glucose skeleton H), 2.89 (–NHC(***H***^a^)(H^b^)(CH_2_)_6_CH_3_, overlapped with water) [29], 2.69 (1H, –NHC(H^a^)(***H***^b^)(CH_2_)_6_CH_3_) [29], 2.29–1.98 (6H, Ph–CH_3_), 1.47–0.92 (12H, –NHC(H^a^)(H^b^)(C***H***_2_)_6_CH_3_), 0.88–0.66 (3H, –NHC(H^a^)(H^b^)(CH_2_)_6_C***H***_3_); Elemental analysis (EA, %): Calculated (C_31_H_42_N_4_O_7_·H_2_O)_n_, C 61.98, H 7.38, N 9.33, Found, C 61.44, H 7.48, N 9.00.

*Chitosan bis(3-chloro-4-methylphenylcarbamate)-(n-octyl urea)* (**CS 2**), yield: 94%; IR (KBr, cm^−1^) υ: 3400, 3325 (–CONH–), 2950, 2850 (–C–H), 1721 (–CO_2_–), 1590 (–Ph), 1657, 1524 (–CONH–); ^1^H-NMR (δ, ppm, 600 MHz, DMSO-*d*_6_, 90 °C): 9.33–8.82 (2H, –NH–CO_2_–), 7.68–6.84 (6H, Ph–H), 5.80–5.29 (2H, –NH–CO–NH–), 5.10–3.20 (7H, glucose skeleton H), ~2.89–2.60 (–NHC(***H***^a^)(***H***^b^)(CH_2_)_6_CH_3_, overlapped with water), 2.32–2.00 (6H, Ph–CH_3_), 1.37–0.69 (15H, –NHC(H^a^)(H^b^)(C***H***_2_)_6_C***H***_3_); EA (%): Calculated (C_31_H_40_Cl_2_N_4_O_7_·1.5H_2_O)_n_, C 54.87, H 6.39, N 8.26, Found, C 54.78, H 6.38, N 7.54.

*Chitosan bis(4-chlorophenylcarbamate)-(n-octyl urea)* (**CS 3**), yield: 90%; IR (KBr, cm^−1^) υ: 3400, 3326 (–CONH–), 2954, 2851 (–C–H), 1721 (–CO_2_–), 1599 (–Ph), 1655, 1532 (–CONH–); ^1^H-NMR (δ, ppm, 600 MHz, DMSO-*d*_6_, 90 °C): 9.33–8.92 (2H, –NH–CO_2_–), 7.55–6.97 (8H, Ph–H), 5.88–5.26 (2H, –NH–CO–NH–), 5.02–~3.48 (glucose skeleton H, overlapped with water), 2.99–2.63 (2H, –NHC(***H***^a^)(***H***^b^)(CH_2_)_6_CH_3_), 1.39–0.95 (12H, –NHC(H^a^)(H^b^)(C***H***_2_)_6_CH_3_), 0.90–0.75 (3H, –NHC(H^a^)(H^b^)(CH_2_)_6_C***H***_3_); EA (%): Calculated (C_29_H_36_Cl_2_N_4_O_7_·1.5H_2_O)_n_, C 53.54, H 6.04, N 8.61, Found, C 53.86, H 6.35, N 7.89.

*Chitosan bis(3,4-dichlorophenylcarbamate)-(n-octyl urea)* (**CS 4**), yield: 94%; IR (KBr, cm^−1^) υ: 3403, 3320 (–CONH–), 2951, 2854 (–C–H), 1724 (–CO_2_–), 1594 (–Ph), 1663, 1521 (–CONH–); ^1^H-NMR (δ, ppm, 600 MHz, DMSO-*d*_6_, 90 °C): 9.45 –9.12 (2H, –NH–CO_2_–), 7.80–7.03 (6H, Ph–H), 5.73–5.20 (2H, –NH–CO–NH–), 5.00–3.28 (7H, glucose skeleton H), ~2.88–~2.55 (–NHC(***H***^a^)(***H***^b^)(CH_2_)_6_CH_3_, overlapped with water and DMSO respectively), 1.34–0.89 (12H, –NHC(H^a^)(H^b^)(C***H***_2_)_6_CH_3_), 0.84 (3H, –NHC(H^a^)(H^b^)(CH_2_)_6_C***H***_3_); EA (%): Calculated (C_29_H_34_Cl_4_N_4_O_7_·2H_2_O)_n_, C 47.82, H 5.26, N 7.69, Found, C 48.52, H 5.30, N 7.01.

*Chitosan bis(3,5-dichlorophenylcarbamate)-(n-octyl urea)* (**CS 5**), yield: 85%; IR (KBr, cm^−1^) υ: 3406, 3323 (–CONH–), 2954, 2854 (–C–H), 1724 (–CO_2_–), 1591 (–Ph), 1666, 1535 (–CONH–); ^1^H-NMR (δ, ppm, 600 MHz, DMSO-*d*_6_, 90 °C): 9.52–9.28 (2H, –NH–CO_2_–), 7.55–6.70 (6H, Ph–H), 5.75–5.24 (2H, –NH–CO–NH–), 5.05–3.37 (7H, glucose skeleton H), ~2.83 (–NHC(***H***^a^)(***H***^b^)(CH_2_)_6_CH_3_, overlapped with water), 1.34–0.91 (12H, –NHC(H^a^)(H^b^)(C***H***_2_)_6_CH_3_), 0.84 (3H, –NHC(H^a^)(H^b^)(CH_2_)_6_C***H***_3_); EA (%): Calculated (C_29_H_34_Cl_4_N_4_O_7_·H_2_O)_n_, C 49.03, H 5.11, N 7.89, Found, C 49.48, H 5.10, N 7.15.

*Chitosan bis(4-trifluoromethoxyphenylcarbamate)-(n-octyl urea)* (**CS 6**), yield: 87%; IR (KBr, cm^−1^) υ: 3326 (–CONH–), 2959, 2856 (–C–H), 1721 (–CO_2_–), 1610 (–Ph), 1652, 1549 (–CONH–); ^1^H-NMR (δ, ppm, 600 MHz, DMSO-*d*_6_, 90 °C): 9.39–8.94(2H, –NH–CO_2_–), 7.57–6.78 (8H, Ph–H), 5.81–5.29 (2H, –NH–CO–NH–), 5.12–3.37 (7H, glucose skeleton H), ~2.85–~2.77 (–NHC(***H***^a^)(***H***^b^)(CH_2_)_6_CH_3_, overlapped with water), 1.38–0.71 (15H, –NHCH_2_(C***H***_2_)_6_C***H***_3_); EA (%): Calculated (C_31_H_36_F_6_N_4_O_9_·0.5H_2_O)_n_, C 50.89, H 5.10, N 7.66, Found, C 51.28, H 5.62, N 7.08.

### 3.3. Swelling Capacity Evaluation of Chitosan Derivatives

About 0.4 g (*w*_0_) of each chiral selector, i.e., **CSs 1**–**6**, was individually immersed in a sample bottle containing 15 mL of ethyl acetate, acetone and tetrahydrofuran (THF). The bottles were sealed and placed in a water bath at 25 °C for 48 h allowing the CS to be swollen thoroughly. The swollen CS was filtered off and weighed (*w*). The swelling capacity was calculated by the formula (*w*–*w*_0_)/*w*_0_.

### 3.4. Preparation of Chiral Stationary Phases and Columns Packing

**CSPs 1**–**6** were prepared by coating **CSs 1**–**6** on macroporous 3-aminopropyl silica gel in a usual manner [36]. Typically, 0.64 g of a CS was dissolved in DMF (30 mL). The resulting solution (10 mL) was mixed with the 3-aminopropyl silica gel (2.56 g), and the DMF was distilled off in vacuum, and the coating was processed twice again with the rest CS solution. After the completion of the coating, the residual solid was dried in vacuum to afford the realted CSP as a white powder. **CSs 1**–**6** were fed at 20% by weight for coating.

**CSPs 1**–**6** were packed into empty chromatographic columns with a slurry method [34], in which the slurries were prepared with a solution of *n*-hexane/isopropanol (90/10), and the displacing solvent was *n*-hexane. The packing pressure was set at 5000 psi. The ADMPC- and CDMPC-based CSPs were prepared in the previous works and packed in the same manner, in which ADMPC and CDMPC were also fed at 20% by weight for coating with the same silica gel as the support [36,37]. The two CSPs were named as ADMPC and CDMPC.

### 3.5. Enantioseparation Evaluation of Chiral Stationary Phases

The column efficiency corresponding to **CSPs 1**–**6** was tested in *n*-hexane/isopropanol (90/10) with 1,1′-biphenyl as the solute. The enantioseparation capability of **CSPs 1**–**6** was evaluated in *n*-hexane/isopropanol (90/10), *n*-hexane/ethanol (90/10) and *n*-hexane/ethanol/methanol (90/5/5) towards the same chiral analytes (Figure 3). The sample solutions were prepared by dissolving the chiral analytes in ethanol at a concerntration of 1 mg/mL and filtered by 0.45 μm filter. During enantioseparation, the solutions were auto-sampled at 10 μL. All chromatographic measurements including column efficiency and enantioseparation capability evaluations were implemented at 25 °C with a flow rate of 1 mL/min. The chiral analytes were measured by a UV detector and the results were recorded at the wavelegth where the analytes absorb most. The retention factor (*k*), separation factor (*α*) and resolution (*R*_s_) were calculated according to the formulas *k* = (*t* − *t*_0_)/*t*_0_, *α* = *k*_2_/*k*_1_ and *R*_s_ = 2(*t*_2_ − *t*_1_)/(*w*_1_ + *w*_2_), in which *t*_1_ and *t*_2_ were, respectively, the retention time of the first- and second-eluted enantiomers, *w*_1_ and *w*_2_ were the peak width of the two enantiomers and the dead time (*t*_0_) was determined with 1,3,5-tris(*tert*-butyl)benzene as the non-retained substance. The elution orders were determined by changing the mass ration of the injected two enantiomers such that at least one enantiomer was avilable, or determined with a polarimeter (PDR-Chiral Inc., Lake Park, FL, USA) if no single enantiomer of a chiral analyte was available.

## 4. Conclusions

The prepared CSPs display satisfactory enantioseparation capability for the tested chiral compounds. Meanwhile, the CSP prepared with chitosan bis(3,5-dichlorophenylcarbamate)-(*n*-octyl urea) (**CSP 5**) that showed the highest swelling capacity in ethyl acetate and THF could safely work in pure ethyl acetate and *n*-hexane/THF (30/70), but would be damaged by pure THF at a low level. The other CSPs, the swelling capacity of which is lower than that of **CSP 5** can also be used with pure ethyl acetate and a normal phase containing a high proportion of THF. One noteworthy point is that the enantioseparation performance can recover after **CSP 5** has been allowed to stand for a period of time without any treatment. It is easily imagined that, during this period, the suprastructure of the chitosan bis(3,5-dichlorophenylcarbamate)-(*n*-octyl urea) changed gradually from one state to another. The latter condition was possibly in a form of lower energy level. It is conceivable that the other chiral selectors prepared in this work also possess this property. If this speculation is true, the enantioseparation capability of a damaged CSP with a polysaccharide derivative as the chiral selector may be recovered just allowing the chiral column to stand for a period of time, provided that the CSP is not seriously damaged.

## Figures and Tables

**Table 1:** Swelling capacity of **CSs 1**–**6** in organic solvents.

Solvent	Swelling Capacity					
	CS 1	CS 2	CS 3	CS 4	CS 5	CS 6
Ethyl acetate	2.5	2.4	2.5	3.2	3.3	2.7
Acetone	1.7	1.2	1.9	1.8	1.6	2.1
THF	9.7	4.6	4.0	11.6	20.2	5.3

**Table 2:** The enantioseparation of **CSP 5** under different conditions.

S.N.	I			II			III			IV			V		
	*k*_1_	*α*	*R*_S_	*k*_1_	*α*	*R*_S_	*k*_1_	*α*	*R*_S_	*k*_1_	*α*	*R*_S_	*k*_1_	*α*	*R*_S_
1	0.34	1.37	1.54	0.34	1.32	1.61	0.34	1.31	1.55	0.30	1.26	1.16	0.31	1.28	1.40
2	0.62	1.00	0.00	0.65	1.00	0.00	0.63	1.00	0.00	0.55	1.00	0.00	0.57	1.00	0.00
3	2.26	1.18	2.08	2.31	1.19	2.20	2.26	1.20	2.24	1.93	1.23	2.18	2.06	1.22	2.25
4	1.26	1.27	3.01	1.29	1.29	2.98	1.25	1.29	2.91	1.05	1.27	2.54	1.16	1.29	2.99
5	3.57	1.16	2.25	3.70	1.18	2.53	3.69	1.17	2.34	3.19	1.16	1.92	3.29	1.17	2.25
6	2.19	1.00	0.00	2.10	1.00	0.00	2.10	1.00	0.00	2.01	1.00	0.00	2.13	1.00	0.00
7	0.47	1.00	0.00	0.41	1.00	0.00	0.43	1.00	0.00	0.41	1.00	0.00	0.42	1.00	0.00
8	2.57	1.00	0.00	2.27	1.00	0.00	2.34	1.00	0.00	2.19	1.00	0.00	2.44	1.00	0.00
9	0.91	1.00	0.00	0.91	1.00	0.00	1.08	1.00	0.00	0.96	1.00	0.00	0.95	1.00	0.00
10	1.38	1.24	2.20	1.46	1.24	2.40	1.44	1.22	2.18	1.28	1.18	1.73	1.37	1.20	2.14
11	2.63	1.14	1.53	2.59	1.12	1.29	2.49	1.13	1.35	2.12	1.16	1.57	2.30	1.16	1.60
12	1.92	2.16	8.46	2.04	2.28	9.14	1.84	2.27	8.85	1.59	2.07	7.41	1.79	2.20	8.71
13	0.93	1.98	9.80	0.98	2.01	10.54	0.95	2.02	10.06	0.83	1.97	7.47	0.87	2.03	8.52
14	2.42	1.28	1.23	2.69	1.25	1.64	2.68	1.25	1.72	2.16	1.23	0.75	2.38	1.24	2.07
15	1.87	2.48	10.65	1.94	2.62	11.74	1.96	2.04	11.85	1.69	2.42	11.03	1.81	2.49	9.06
16	1.44	2.08	6.33	1.58	2.16	6.71	1.51	2.09	6.52	1.32	1.96	5.21	1.39	2.06	6.75
17	1.65	1.14	2.09	1.77	1.13	2.00	1.74	1.11	1.69	1.54	1.08	1.20	1.66	1.09	1.16
18	11.07	1.10	1.32	11.21	1.10	1.34	10.74	1.10	1.15	8.66	1.10	1.05	9.56	1.11	1.28
19	10.90	1.48	3.87	11.36	1.61	5.61	10.61	1.58	5.37	9.01	1.45	4.07	10.19	1.45	3.72
20	3.85	5.83	19.50	3.99	5.85	19.96	3.81	5.77	19.23	3.23	5.43	17.35	3.54	5.76	19.07

S.N.: serial number of chiral compounds. Eluent: *n*-hexane/ethanol (90/10). The conditions of **CSP 5**: I: after **CSP 5** had been analyzed with *n*-hexane/isopropanol (90/10), *n*-hexane/ethanol (90/10) and *n*-hexane/methanol/ethanol (90/5/5), and then was allowed to stand for one month; II: after **CSP 5** had been analyzed in condition I and then flushed with pure ethyl acetate; III: after **CSP 5** had been analyzed in condition II and then flushed with *n*-hexane/THF (30/70); IV: after **CSP 5** had been analyzed in condition III and then flushed with pure THF; V: after **CSP 5** had been analyzed in condition IV and was then allowed to stand for two months. Column size: Φ 250 mm × 4.6 mm; flow rate: 1 mL/min; detection temperature: 25 °C.

## Supplementary Materials

The following are available online at http://www.mdpi.com/1420-3049/21/11/1528/s1.

## References

[1] Chai T., Yang W., Qiu J., Hou S. (2015). Direct Enantioseparation of nitrogen-heterocyclic pesticides on cellulose-based chiral column by high-performance liquid chromatography. Chirality.

[2] Geryk R., Kalíkova K., Schmid M.G., Tesarova E. (2016). Enantioselective separation of biologically active basic compounds in ultra-performance supercritical fluid chromatography. Anal. Chim. Acta.

[3] Batra S., Bhushan R. (2016). Resolution of enantiomers of bupropion and its metabolites by liquid chromatography. Biomed. Chromatogr..

[4] Skogsberg U., Allenmark S. (2001). Some conformationally restricted chiral stationary phase selector units related to *N*,*N*′-diallyl-l-tartardiamide. Chromatographia.

[5] Thunberg L., Allenmark S. (2004). Resolution studies on two regioisomeric chiral stationary phases: Effects from reversed orientation of an amide group. J. Chromatogr. A.

[6] Shen J., Okamoto Y. (2016). Efficient Separation of enantiomers using stereoregular chiral polymers. Chem. Rev..

[7] Wu D.-R., Yip S.H., Li P., Sun D., Mathur A. (2016). From analytical methods to large scale chiral SFC using chlorinated chiral stationary phases. J. Chromatogr. A.

[8] Zhou J., Chen S., Luo P., Sun C., Meng L., Du Q., Sun F. (2015). Chiral separations in normal-phase liquid chromatography: updating a screening strategy with a chlorine-containing polysaccharide-based selector. J. Chin. Chem. Soc..

[9] Sharp V.S., Gokey M.A., Wolfe C.N., Rener G.A., Cooper M.R. (2015). High performance liquid chromatographic enantioseparation development and analytical method characterization of the carboxylate ester of evacetrapib using an immobilized chiral stationary phase with a non-conventional eluent system. J. Chromatogr. A.

[10] Zhang X., Wang L., Dong S., Zhang X., Wu Q., Zhao L., Shi Y. (2016). Nanocellulose 3,5-dimethylphenylcarbamate derivative coated chiral stationary phase: Preparation and enantioseparation performance. Chirality.

[11] Sun L., Wei P., Ouyang P., Zou Q. (2016). Chiral separation and thermodynamic investigation of ezetimibe optical isomers on a chiralpak IC column. J. Chromatogr. Sci..

[12] Rahou I., Sekkoum K., Belboukhari N., Cheriti A., Aboul-Enein H.Y. (2016). Liquid chromatographic separation of novel 4-amino-flavanes series diastereomers on a polysaccharide-type chiral stationary phase. J. Chromatogr. Sci..

[13] Tan Y., Fan J., Lin C., Tu H., Zheng S., Zhang W. (2014). Synthesis and enantioseparation behaviors of novel immobilized 3,5-dimethylphenylcarbamoylated polysaccharide chiral stationary phases. J. Sep. Sci..

[14] Thurmann S., Lotter C., Heiland J.J., Chankvetadze B., Belder D. (2015). Chip-based high-performance liquid chromatography for high-speed enantioseparations. Anal. Chem..

[15] Khater S., West C. (2014). Insights into chiral recognition mechanisms in supercritical fluid chromatography V. Effect of the nature and proportion of alcohol mobile phase modifier with amylose and cellulose tris-(3,5-dimethylphenylcarbamate) stationary phases. J. Chromatogr. A.

[16] Ates H., Younes A.A., Mangelings D., Heyden Y.V. (2013). Enantioselectivity of polysaccharide-based chiral selectors in polar organic solvents chromatography: Implementation of chlorinated selectors in a separation strategy. J. Pharmaceut. Biomed. Anal..

[17] Rao R.N., Kumar K.N., Naidu C.G. (2012). Liquid chromatographic separation of darunavir enantiomers on coated and immobilized amylose tris(3,5-dimethylphenylcarbamate) chiral stationary phases. Chirality.

[18] Okamoto Y., Ikai T., Shen J. (2011). Controlled immobilization of polysaccharide derivatives for efficient chiral separation. Isr. J. Chem..

[19] Ikai T., Yamamoto C., Kamigaito M., Okamoto Y. (2008). Immobilized-type chiral packing materials for HPLC based on polysaccharide derivatives. J. Chromatogr. B.

[20] Francotte E., Zhang T. (2016). Preparation and evaluation of immobilized 4-methylbenzoylcellulose stationary phases for enantioselective separations. J. Chromatogr. A.

[21] Yamamoto C., Hayashi T., Okamoto Y. (2003). High-performance liquid chromatographic enantioseparation using chitin carbamate derivatives as chiral stationary phases. J. Chromatogr. A.

[22] Zhang L., Shen J., Zuo W., Okamoto Y. (2014). Synthesis of chitosan 3,6-diphenylcarbamate-2-urea derivatives andtheir applications as chiral stationary phases for high-performanceliquid chromatography. J. Chromatogr. A.

[23] Yamamoto C., Fujisawa M., Kamigaito M., Okamoto Y. (2008). Enantioseparation using urea- and imide- bearing chitosan phenylcarbamate derivatives as chiral stationary phases for high-performance liquid chromatography. Chirality.

[24] Tang S., Bin Q., Chen W., Bai Z.-W., Huang S.-H. (2016). Chiral stationary phases based on chitosanbis(methylphenylcarbamate)-(isobutyrylamide) forhigh-performance liquid chromatography. J. Chromatogr. A.

[25] Zhang J., Wang X.-C., Chen W., Bai Z.-W. (2016). Synthesis of substituted phenylcarbamates of *N*-cyclobutylformylated chitosan and their application as chiral selectors in enantioseparation. Analyst.

[26] Feng Z.-W., Huang S.-H., Chen W., Bai Z.-W. (2016). Selective and full derivatization of amino group in chitosan with alkyl chloroformate of low stereo-hindrance. Macromol. Res..

[27] Feng Z.-W., Chen W., Bai Z.-W. (2016). Chiral stationary phases based on chitosan bis(4-methylphenylcarbamate)-(alkoxyformamide). J. Sep. Sci..

[28] Wang J., Jiang J.-Z., Chen W., Bai Z.-W. (2016). Synthesis and characterization of chitosan alkyl urea. Carbohydr. Polym..

[29] Wang J., Xi J.-B., Chen W., Huang S.-H., Bai Z.-W. (2017). High performance chiral separation materials based on chitosan bis(3,5-dimethylphenylcarbamate)-(alkyl urea)s. Carbohydr. Polym..

[30] Shen J., Li G., Yang Z., Okamoto Y. (2016). Synthesis and chiral recognition of amylose derivatives bearing regioselective phenylcarbamate substituents at 2,6- and 3-positions for high-performance liquid chromatography. J. Chromatogr. A.

[31] Ma S., Shen S., Lee H., Eriksson M., Zeng X., Xu J., Fandrick K., Yee N., Senanayake C., Grinberg N. (2009). Mechanistic studies on the chiral recognition of polysaccharide-based chiral stationary phases using liquid chromatography and vibrational circular dichroism. J. Chromatogr. A.

[32] Mima S., Miya M., Iwamoto R., Yoshikawa S. (1983). Highly deacetylated chitosan and its properties. J. Appl. Polym. Sci..

[33] Tolaimate A., Desbrieres J., Rhazi M., Alagui A. (2003). Contribution to the preparation of chitins and chitosans with controlled physico-chemical properties. Polymer.

[34] Wei W.-J., Deng H.-W., Chen W., Bai Z.-W., Li S.-R. (2010). Preparation and enantioseparation of a mixed selector chiral stationary phase derived from benzoylated tartaric acid and 1,2-diphenylethylenediamine. Chirality.

[35] Huang S.-H., Bai Z.-W., Feng J.-W. (2009). Chiral self-discrimination of the enantiomers of α-phenylethylamine derivatives in proton NMR. Magn. Reson. Chem..

[36] Wang Z.-Q., Liu J.-D., Chen W., Bai Z.-W. (2014). Enantioseparation characteristics of biselector chiral stationary phases based on derivatives of cellulose and amylase. J. Chromatogr. A.

[37] Wang X.-C., Zhang J., Xu X.-Q., Chen W., Yang Y.-G., Bai Z.-W. (2015). Enantioseparation characteristics of chiral stationary phases based on the derivatives of cellulose and chitin. Anal. Methods.

