# Supplementary material for: Eluent Tolerance and Enantioseparation Recovery of Chiral Packing Materials Based on Chitosan Bis(Phenylcarbamate)-(n-Octyl Urea)s for High Performance Liquid Chromatography
Source: Molecules. 2016 Nov 13;21(11):1528. doi: 10.3390/molecules21111528 (PMC6272896; doi:10.3390/molecules21111528)
Supplement: Supplementary file 1 [file molecules-21-01528-s001.pdf]

Eluent Tolerance and Enantioseparation Recovery of Chiral Packing Materials Based on Chitosan Bis(Phenylcarbamate)-(*n*-Octyl Urea)s for High Performance Liquid Chromatography

Jing Wang, Shao-Hua Huang, Wei Chen and Zheng-Wu Bai

1. ^{1}H-NMR Spectra Of Chitosan and Chitosan Derivatives

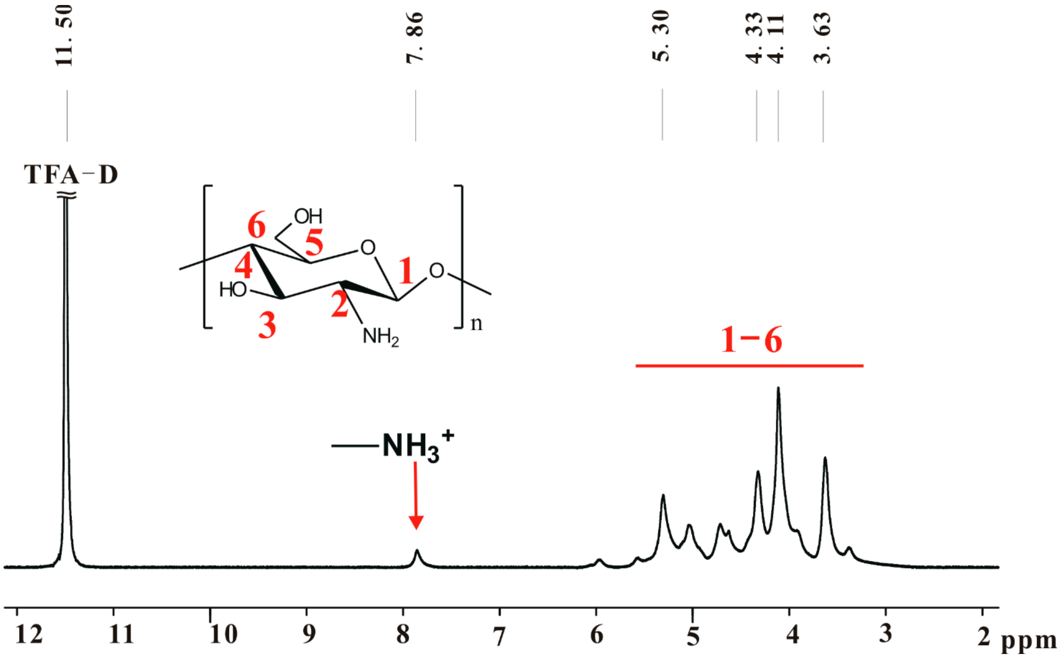

Figure S1. ^{1}H-NMR spectrum of chitosan (Ia) with the M_v of 1.5 × 10^5.

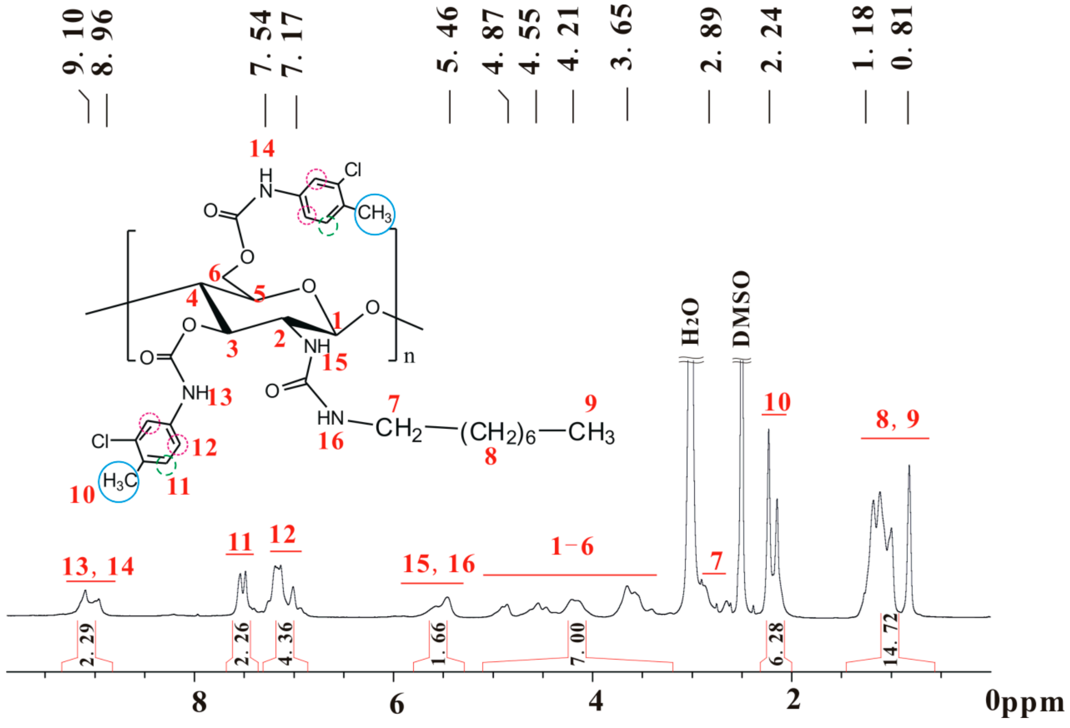

Figure S2. ^{1}H-NMR spectrum of **CS 2**.

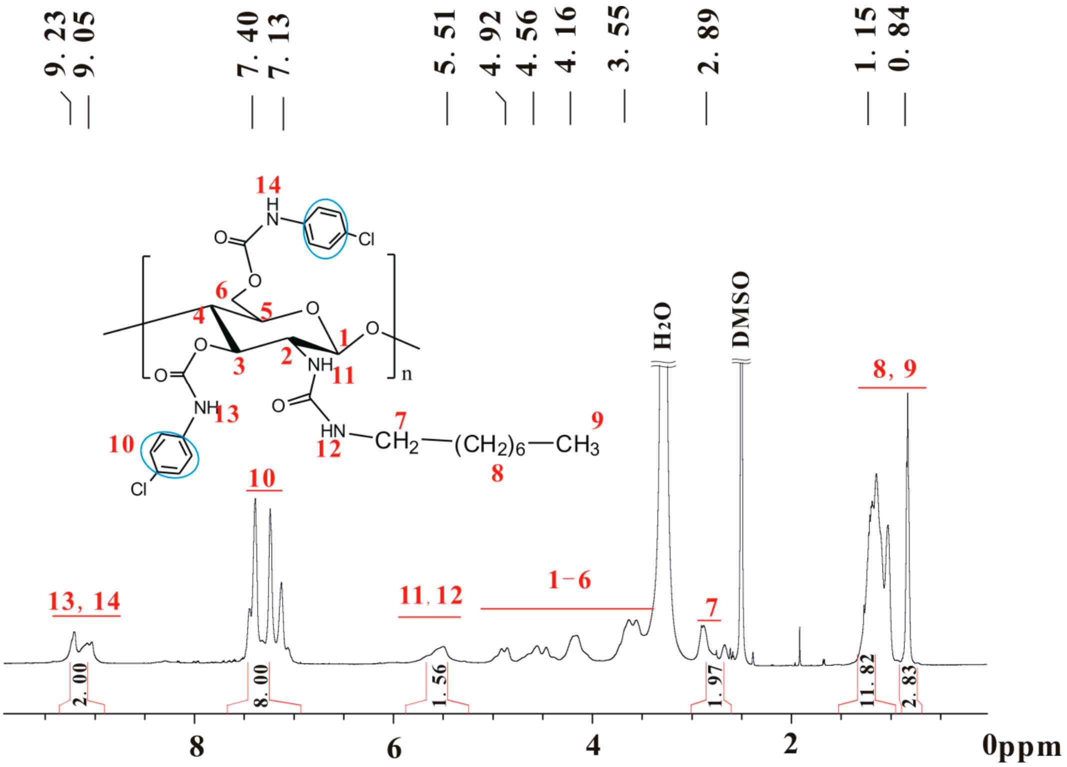

Figure S3. ^{1}H-NMR spectrum of **CS 3**.

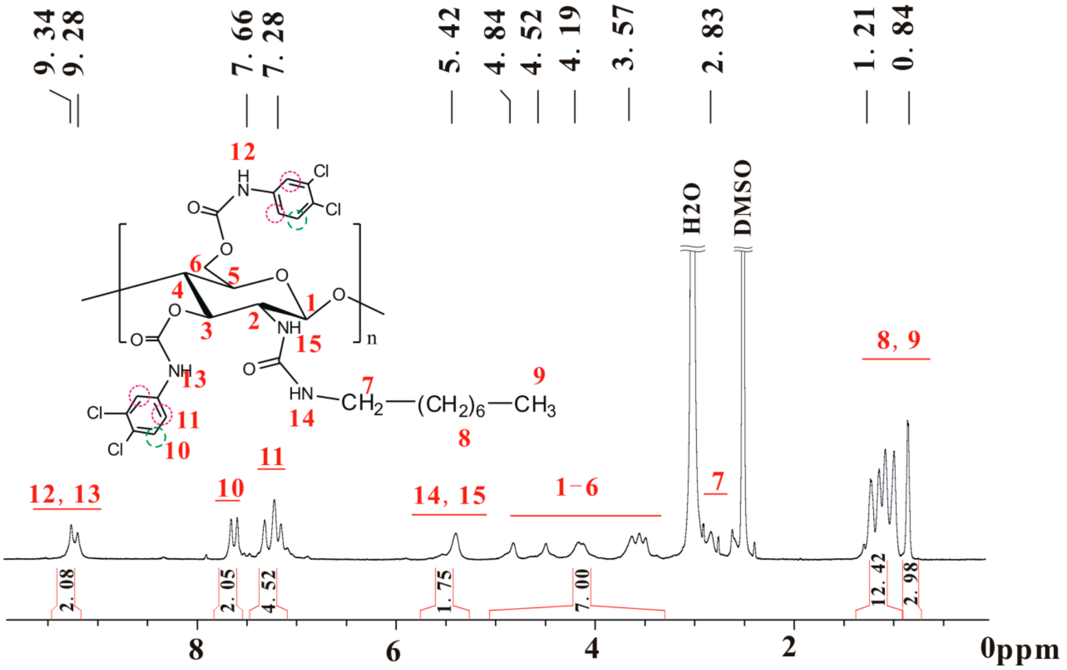

Figure S4. ^{1}H-NMR spectrum of **CS 4**.

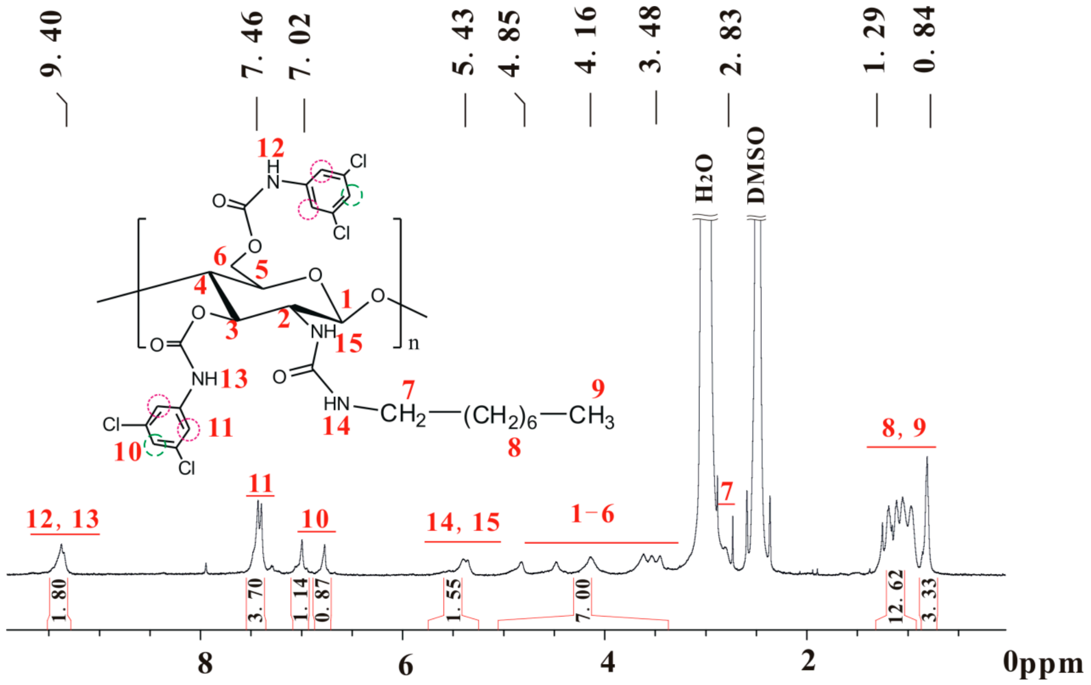

Figure S5. ¹H-NMR spectrum of **CS 5**.

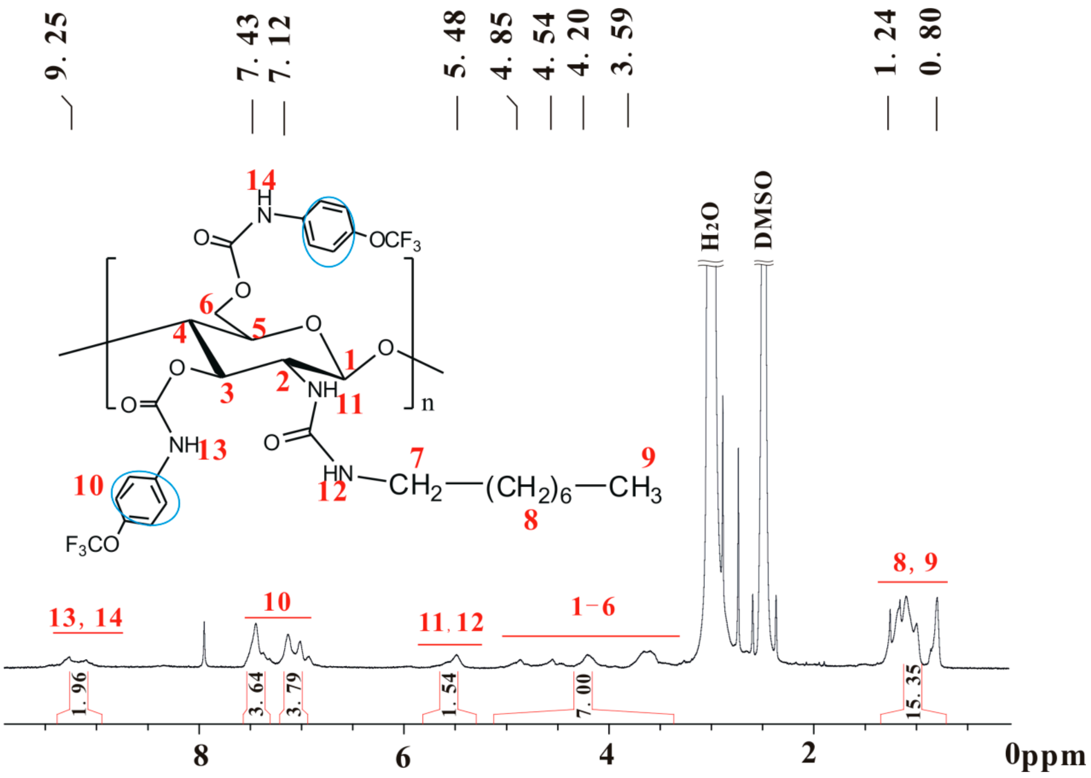

Figure S6. ¹H-NMR spectrum of **CS 6**.

2. IR Spectra of Chitosan Derivatives

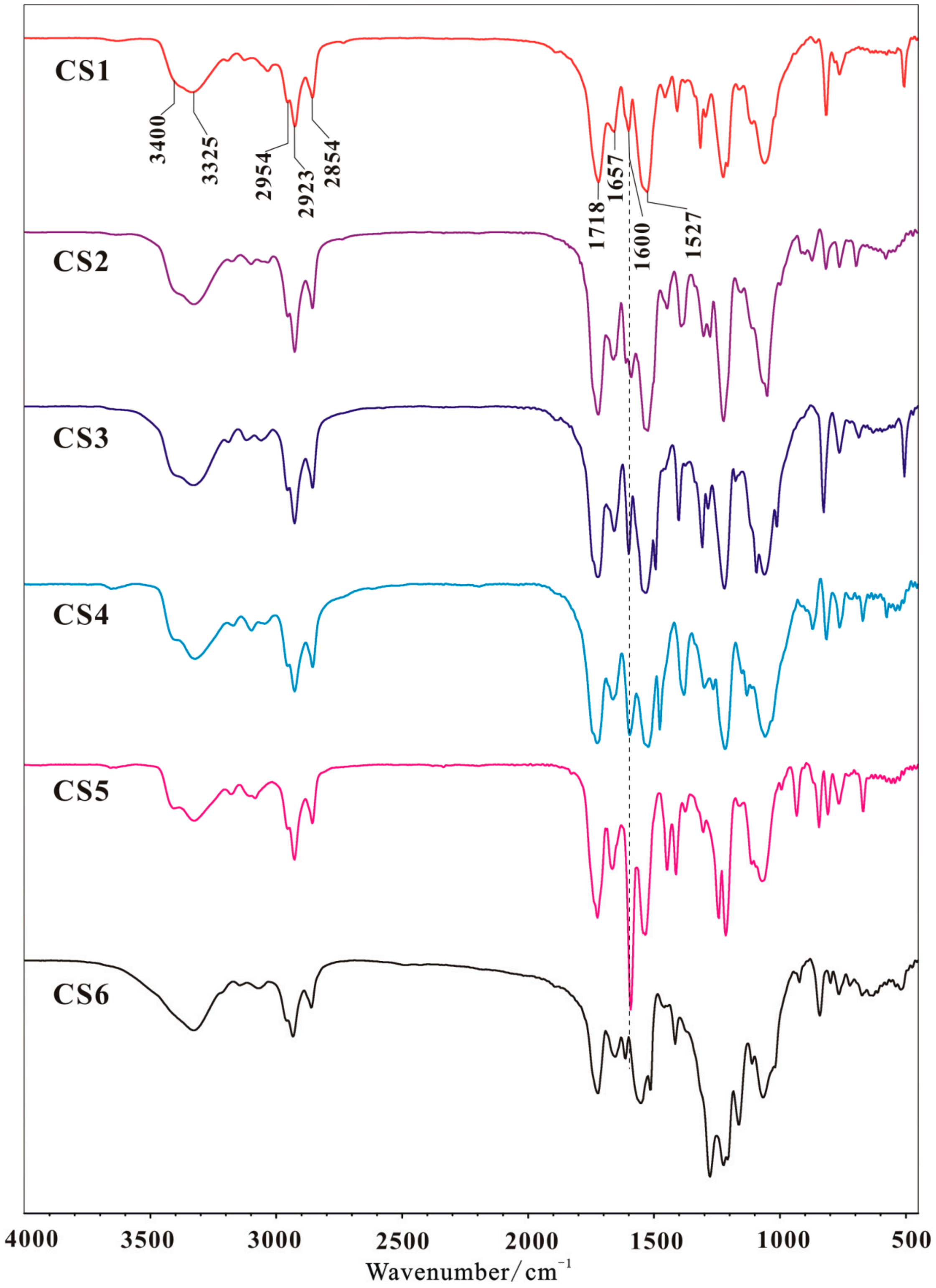

Figure S7. IR spectra of **CSs 1–6**.

3. Enantioseparation Evaluation of CSPs 1–6, ADMPC and CDMPC

Table S1. Enantioseparation evaluation of CSPs **1**–**6**.

S.N.	CSP 1			CSP 2			CSP 3			CSP 4			CSP 5			CSP 6			M.P.
	k_1	α	R_s	k_1	α	R_s	k_1	α	R_s	k_1	α	R_s	k_1	α	R_s	k_1	α	R_s	
**1**	$^{-}$0.27	1.09	0.46	$^{-}$0.25	1.77	2.12	$^{-}$0.29	1.75	3.21	$^{-}$0.43	1.53	2.63	0.44	1.00	0.00	$^{-}$0.21	1.60	1.67	A
	$^{-}$0.24	1.11	0.54	$^{-}$0.20	1.68	1.99	$^{-}$0.27	1.55	2.45	$^{-}$0.37	1.51	2.51	$^{-}$0.34	1.37	1.54	$^{-}$0.20	1.38	0.75	B
	$^{-}$0.28	1.39	1.81	$^{-}$0.22	1.50	1.60	$^{-}$0.29	1.38	1.76	$^{-}$0.39	1.33	1.64	$^{-}$0.40	1.23	1.37	$^{-}$0.23	1.18	0.51	C
**2**	1.56	1.00	0.00	1.10	1.00	0.00	0.90	1.00	0.00	0.88	1.00	0.00	0.92	1.00	0.00	0.66	1.00	0.00	A
	0.97	1.00	0.00	0.65	1.00	0.00	0.67	1.00	0.00	0.70	1.00	0.00	0.62	1.00	0.00	0.49	1.00	0.00	B
	0.93	1.00	0.00	0.61	1.00	0.00	0.64	1.00	0.00	0.76	1.00	0.00	0.67	1.00	0.00	0.63	1.00	0.00	C
**3**	$^{+}$2.36	1.05	0.61	$^{+}$2.33	1.08	0.58	$^{-}$2.56	1.14	1.45	$^{+}$4.70	1.04	0.55	$^{-}$3.38	1.31	3.61	$^{-}$2.03	1.16	1.04	A
	$^{+}$1.80	1.08	1.04	1.64	1.00	0.00	$^{+}$1.87	1.10	1.38	$^{-}$2.95	1.00	0.00	$^{-}$2.26	1.18	2.08	$^{-}$1.29	1.29	2.96	B
	$^{+}$1.77	1.26	2.44	1.52	1.00	0.00	$^{+}$1.60	1.14	1.47	$^{-}$2.52	1.00	0.00	$^{-}$2.02	1.12	1.42	$^{-}$1.23	1.31	2.79	C
**4**	$^{-}$0.95	1.60	5.38	$^{-}$1.13	1.36	2.73	$^{-}$1.04	1.42	3.92	$^{-}$2.67	1.28	2.68	$^{-}$1.96	1.29	3.65	$^{-}$0.71	1.48	2.32	A
	$^{-}$0.82	1.72	6.61	$^{-}$0.73	1.41	2.83	$^{-}$0.77	1.42	3.81	$^{-}$1.65	1.25	3.08	$^{-}$1.26	1.27	3.01	$^{-}$0.47	1.40	1.88	B
	$^{-}$0.92	1.57	5.80	$^{-}$0.70	1.32	2.29	$^{-}$0.69	1.33	2.93	$^{-}$1.35	1.21	2.29	$^{-}$1.18	1.15	1.52	$^{-}$0.49	1.35	2.06	C
**5**	$^{+}$2.41	1.27	3.30	$^{+}$3.27	1.19	1.50	$^{+}$3.74	1.20	2.77	$^{+}$8.57	1.11	1.68	$^{+}$7.42	1.20	2.80	$^{+}$3.30	1.29	2.36	A
	$^{+}$1.35	1.25	2.84	$^{+}$1.61	1.15	1.11	$^{+}$2.06	1.18	2.21	$^{+}$3.98	1.11	1.49	$^{+}$3.57	1.16	2.25	$^{+}$1.61	1.20	1.35	B
	$^{+}$1.24	1.20	2.43	$^{+}$1.35	1.05	0.46	$^{+}$1.60	1.08	1.08	$^{+}$2.81	1.07	0.99	$^{+}$2.71	1.10	1.35	$^{+}$1.51	1.10	1.01	C
**6**	$^{-}$3.92	1.15	1.28	$^{-}$3.28	0.16	0.46	$^{-}$3.42	1.03	0.3	4.14	1.00	0.00	3.13	1.00	0.00	2.61	1.00	0.00	A
	$^{-}$2.52	1.05	0.45	1.96	1.00	0.00	2.34	1.00	0.00	2.63	1.00	0.00	2.19	1.00	0.00	1.77	1.00	0.00	B
	2.18	1.00	0.00	1.64	1.00	0.00	1.86	1.00	0.00	2.22	1.00	0.00	1.75	1.00	0.00	1.69	1.00	0.00	C
**7**	0.72	1.00	0.00	0.44	1.00	0.00	0.35	1.00	0.00	0.38	1.00	0.00	0.41	1.00	0.00	0.28	1.00	0.00	A
	0.57	1.00	0.00	0.38	1.00	0.00	0.43	1.00	0.00	0.51	1.00	0.00	0.47	1.00	0.00	0.34	1.00	0.00	B
	0.63	1.00	0.00	0.46	1.00	0.00	0.48	1.00	0.00	0.59	1.00	0.00	0.50	1.00	0.00	0.46	1.00	0.00	C
**8**	^{S}1.09	1.90	3.10	^{S}1.01	1.24	1.15	^{S}1.14	1.06	0.50	^{S}3.26	1.07	0.74	^{S}3.70	1.15	0.51	4.01	1.16	0.44	A
	^{S}0.88	1.47	2.05	0.71	1.00	0.00	1.70	1.00	0.00	2.35	1.00	0.00	2.57	1.00	0.00	2.31	1.00	0.00	B
	^{S}0.92	1.23	1.76	0.76	1.00	0.00	1.28	1.00	0.00	2.09	1.00	0.00	2.10	1.00	0.00	2.15	1.00	0.00	C
**9**	0.80	1.00	0.00	0.76	1.00	0.00	0.83	0.26	0.39	1.24	1.00	0.00	1.71	1.00	0.00	1.78	1.00	0.00	A
	0.51	1.00	0.00	0.46	1.00	0.00	1.08	1.00	0.00	0.91	1.00	0.00	0.91	1.00	0.00	3.07	1.00	0.00	B
	0.53	1.00	0.00	0.49	1.00	0.00	0.82	1.00	0.00	^{R}0.96	1.05	0.54	0.89	1.00	0.00	2.38	1.00	0.00	C
**10**	2.75	1.00	0.00	^{S}2.38	2.16	5.01	^{S}3.41	1.54	2.39	^{S}5.38	1.25	1.86	^{S}3.73	1.46	2.28	^{S}2.48	2.08	2.34	A
	^{R}0.98	1.34	2.10	^{S}0.97	1.35	2.13	^{S}1.55	1.15	1.58	2.17	1.00	0.00	^{S}1.38	1.24	2.20	^{S}1.21	1.13	1.02	B
	^{R}0.85	1.18	1.51	0.88	1.00	0.00	1.18	1.00	0.00	^{R}1.79	1.09	0.99	1.26	1.00	0.00	1.20	1.00	0.00	C
**11**	1.36	1.00	0.00	2.20	1.00	0.00	3.27	1.00	0.00	§6.31	1.56	5.42	6.10	1.00	0.00	2.06	1.00	0.00	A
	0.72	1.00	0.00	1.14	1.00	0.00	1.66	1.00	0.00	§2.87	1.31	3.01	§2.63	1.14	1.53	1.03	1.00	0.00	B
	0.79	1.00	0.00	1.01	1.00	0.00	1.35	1.00	0.00	§2.24	1.26	2.37	§2.02	1.16	1.57	1.07	1.00	0.00	C

Table S1. *Cont.*

S.N.	CSP 1			CSP 2			CSP 3			CSP 4			CSP 5			CSP 6			M.P.
	k_1	α	R_s	k_1	α	R_s	k_1	α	R_s	k_1	α	R_s	k_1	α	R_s	k_1	α	R_s	
**12**	^{R}9.97	1.39	2.78	^{S}10.96	1.22	1.06	^{S}7.80	1.20	1.19	^{S}7.17	1.63	3.33	^{S}3.87	1.71	3.97	^{S}3.68	1.41	2.66	A
	^{S}4.77	1.15	1.54	^{S}4.22	1.61	3.03	^{S}4.13	1.40	3.58	^{S}3.19	2.00	4.10	^{S}1.92	2.16	8.46	^{S}1.71	1.46	3.15	B
	^{S}3.40	1.09	1.12	^{S}2.78	1.38	2.66	^{S}2.64	1.26	2.69	^{S}2.63	1.35	3.29	^{S}1.45	1.61	5.09	^{S}1.63	1.26	2.07	C
**13**	^{S}2.81	1.09	0.88	1.88	1.00	0.00	1.88	1.00	0.00	^{R}1.97	1.31	3.03	^{R}1.56	1.44	3.37	1.45	1.00	0.00	A
	^{R}1.56	1.06	0.56	^{R}1.08	1.14	1.06	^{R}1.25	1.06	0.55	^{R}1.28	1.40	3.40	^{R}0.93	1.98	9.80	0.91	1.00	0.00	B
	^{R}1.37	1.12	1.31	^{R}0.93	1.22	1.58	^{R}1.03	1.07	0.61	^{R}1.18	1.36	3.21	^{R}0.88	2.36	6.92	0.92	1.00	0.00	C
**14**	$^{+}$5.22	1.26	2.12	$^{+}$4.57	1.17	1.16	$^{+}$3.34	1.08	0.52	$^{+}$6.58	1.07	0.58	$^{+}$5.37	1.43	3.09	2.33	1.00	0.00	A
	$^{+}$2.16	1.07	0.55	2.08	1.00	0.00	1.66	1.00	0.00	$^{-}$3.00	1.18	0.98	$^{+}$2.42	1.28	1.23	1.20	1.00	0.00	B
	1.75	1.00	0.00	1.40	1.00	0.00	1.28	1.00	0.00	$^{-}$2.01	1.20	2.16	1.91	1.00	0.00	1.15	1.00	0.00	C
**15**	^{S}13.99	1.11	0.65	7.61	1.00	0.00	^{S}9.49	3.09	8.29	^{S}9.25	2.90	7.82	^{S}7.82	3.58	7.45	^{S}8.05	3.79	8.50	A
	^{S}2.93	1.04	0.40	^{S}1.74	2.61	8.87	^{S}2.56	1.97	6.74	^{S}2.53	2.08	3.11	^{S}1.87	2.48	10.65	^{S}1.64	2.16	6.79	B
	^{S}1.79	1.08	0.70	^{S}1.26	1.40	3.03	^{S}1.70	1.25	2.77	^{S}2.20	1.09	1.02	^{S}1.51	1.23	1.74	^{S}1.51	1.21	2.09	C
**16**	^{S}9.45	1.11	0.76	4.95	1.00	0.00	^{S}5.79	2.94	7.61	^{S}5.76	2.60	6.06	^{S}6.24	2.89	6.62	^{S}5.25	3.72	8.67	A
	1.99	1.00	0.00	^{S}1.22	2.38	6.30	^{S}1.72	1.73	4.07	^{S}1.79	1.75	4.25	^{S}1.44	2.08	6.33	^{S}1.29	1.58	1.64	B
	1.24	1.00	0.00	^{S}0.89	1.39	2.63	^{S}1.22	1.19	1.87	^{S}1.50	1.15	1.49	^{S}1.08	1.31	2.01	^{S}1.18	1.16	1.29	C
**17**	^{R}2.41	1.35	3.27	^{S}2.26	2.42	4.62	^{S}3.65	1.52	2.87	^{S}6.60	1.19	1.52	^{S}4.88	1.34	2.63	^{S}3.18	1.76	1.99	A
	^{R}0.83	1.22	1.57	^{S}1.05	1.39	2.72	^{S}1.60	1.11	1.29	2.65	1.00	0.00	^{S}1.65	1.14	2.09	^{S}1.31	1.10	0.74	B
	^{R}0.75	1.12	1.03	0.89	1.00	0.00	^{R}1.17	1.06	0.58	^{R}1.91	1.09	1.32	1.36	1.00	0.00	1.19	1.00	0.00	C
**18**	^{R}14.77	1.48	4.39	^{R}10.56	1.09	0.63	^{S}11.33	1.15	1.20	^{S}19.48	1.23	2.11	^{S}16.57	1.15	1.65	^{S}8.34	1.23	1.34	A
	^{R}8.21	2.24	9.27	^{R}5.54	1.28	1.96	^{S}7.24	1.02	0.36	^{S}11.58	1.03	0.42	^{R}11.07	1.10	1.32	^{S}4.48	1.14	0.86	B
	^{R}6.53	1.93	8.37	^{R}4.20	1.24	1.92	^{S}4.88	1.08	0.96	^{S}8.37	1.06	0.66	^{R}8.05	1.14	1.66	^{S}3.48	1.16	0.71	C
**19**	^{S}15.59	1.16	0.92	^{S}22.18	1.39	1.71	Retention time > 120min			Retention time > 120min			Retention time > 120min			Retention time > 120min			A
	^{S}5.64	1.13	1.02	^{S}7.51	1.39	2.14	^{S}10.97	1.30	2.26	^{S}15.23	1.32	3.31	^{S}10.90	1.48	3.87	^{S}6.35	1.42	3.26	B
	4.07	1.00	0.00	^{S}5.34	1.21	1.51	^{S}6.32	1.18	1.84	^{S}8.88	1.12	1.30	^{S}7.00	1.17	2.18	^{S}4.31	1.22	2.04	C
**20**	Retention time > 120 min			‡5.79	3.15	9.31	‡4.26	2.54	6.76	‡8.29	3.83	14.75	Retention time > 120min			‡2.57	1.95	3.08	A
	‡3.96	3.25	13.26	‡2.40	2.32	7.35	‡1.96	1.98	8.89	‡3.49	2.45	10.69	‡3.85	5.83	19.50	‡1.33	1.22	2.20	B
	‡2.74	2.37	10.12	‡1.69	1.75	5.20	‡1.56	1.42	4.40	‡2.36	1.62	5.73	‡2.44	3.93	16.41	‡1.30	1.13	1.09	C

S.N.: serial number of the analytes; M.P. (mobile phase): A: *n*-hexane/isopropanol (90/10), B: *n*-hexane/ethanol (90/10), C: *n*-hexane/ethanol/methanol (90/5/5); flow rate: 1 ml/min; detection temperature: 25 °C. "+", "−", "R", "S" and "‡2S,3R", "§4R,1S" at the superscript of k_1 refer to the optical rotation or configuration of the first-eluted enantiomer.

Table S2. Chiral separation results of ADMPC and CDMPC.

S.N.	ADMPC			CDMPC			M.P.
	k_1	α	Rs	k_1	α	Rs	
1	$^{+}$0.65	1.51	1.68	$^{+}$1.03	1.49	2.12	A
	$^{+}$0.54	1.87	3.07	$^{+}$0.70	1.64	2.62	B
	$^{+}$0.66	1.79	3.79	$^{+}$0.72	1.64	2.63	C
2	1.19	1.00	0.00	2.09	1.00	0.00	A
	$^{+}$0.68	1.06	0.28	1.34	1.00	0.00	B
	0.98	1.00	0.00	1.24	1.00	0.00	C
3	3.99	1.00	0.00	$^{+}$2.90	1.40	2.17	A
	$^{+}$2.60	1.08	0.48	$^{+}$2.06	1.33	1.93	B
	$^{+}$2.76	1.10	0.85	$^{+}$1.85	1.34	1.96	C
4	$^{+}$1.24	1.20	1.37	$^{-}$1.53	1.30	1.80	A
	$^{+}$1.19	1.46	2.94	$^{-}$1.15	1.21	1.14	B
	$^{+}$1.43	1.79	5.00	$^{-}$1.09	1.21	1.19	C
5	$^{-}$2.95	1.06	0.48	$^{+}$3.74	1.10	0.57	A
	1.71	1.00	0.00	1.90	1.00	0.00	B
	2.29	1.00	0.00	1.60	1.00	0.00	C
6	2.38	1.00	0.00	2.18	1.00	0.00	A
	1.78	1.00	0.00	1.60	1.00	0.00	B
	1.73	1.00	0.00	1.67	1.00	0.00	C
7	0.63	1.00	0.00	$^{+}$0.95	1.12	0.42	A
	0.43	1.00	0.00	$^{+}$0.65	1.13	0.22	B
	0.69	1.00	0.00	$^{+}$0.57	1.13	0.38	C
8	1.65	1.00	0.00	2.49	1.00	0.00	A
	1.46	1.00	0.00	1.37	1.00	0.00	B
	1.95	1.00	0.00	1.23	1.00	0.00	C
9	^{S}0.89	1.09	0.49	1.06	1.00	0.00	A
	0.75	1.00	0.00	0.75	1.00	0.00	B
	0.75	1.00	0.00	0.83	1.00	0.00	C
10	^{R}2.66	1.42	2.42	^{R}3.16	1.38	1.96	A
	^{R}1.42	1.34	1.89	^{R}1.35	1.26	1.30	B
	^{R}1.13	1.28	1.60	^{R}1.34	1.17	0.90	C
11	^{4R,1S}2.69	2.36	4.81	2.97	1.00	0.00	A
	^{4R,1S}1.65	1.89	3.73	^{4S,1R}1.66	2.36	4.75	B
	^{4R,1S}1.47	1.61	3.13	^{4S,1R}1.86	2.06	4.36	C
12	^{S}7.12	1.07	0.47	9.77	1.00	0.00	A
	^{S}4.37	1.09	0.58	5.61	1.00	0.00	B
	^{S}3.14	1.08	0.57	^{S}3.78	1.08	0.51	C
13	^{S}1.14	1.22	0.84	^{R}1.62	1.34	1.60	A
	^{S}0.58	1.12	0.32	^{R}0.83	1.34	1.55	B
	0.97	1.00	0.00	^{R}0.73	1.21	0.47	C
14	$^{+}$2.42	1.57	2.45	5.71	1.00	0.00	A
	$^{+}$1.37	2.98	6.28	1.75	1.00	0.00	B
	$^{+}$1.72	2.87	6.98	1.36	1.00	0.00	C
15	^{R}10.01	1.45	1.75	^{S}11.74	1.20	0.96	A
	^{R}1.97	1.28	1.33	^{S}2.25	1.12	0.38	B
	^{R}2.36	1.21	1.47	^{S}2.50	1.10	0.35	C
16	^{R}6.11	2.09	3.00	^{R}7.61	1.25	0.39	A
	^{R}1.32	1.41	1.60	^{R}1.42	1.28	1.19	B
	^{R}1.57	1.21	1.37	^{R}1.05	1.20	0.89	C
17	^{R}5.16	1.70	3.15	^{R}4.52	1.18	1.01	A
	^{R}1.22	1.46	2.04	^{R}1.15	1.12	0.53	B
	^{R}1.58	1.27	1.45	^{R}1.10	1.16	0.54	C
18	^{R}5.71	1.27	1.46	^{S}9.85	1.09	0.96	A
	^{R}3.23	1.11	0.77	4.89	1.00	0.00	B
	4.10	1.00	0.00	^{R}3.82	1.10	0.45	C

Table S2. *Cont.*

S.N.	ADMPC			CDMPC			M.P.
	k_1	α	Rs	k_1	α	Rs	
19	Retention time>120 min			^{R}17.56	1.28	0.43	A
	^{R}10.37	1.61	2.57	^{R}6.37	1.25	0.99	B
	^{R}9.62	1.79	3.63	^{R}3.94	1.15	0.54	C
20	^{2R,3S}5.62	1.16	0.78	19.21	1.00	0.00	A
	^{2R,3S}4.65	1.18	1.11	^{2R,3S}9.47	1.29	1.81	B
	^{2R,3S}3.81	1.17	1.16	^{2R,3S}6.53	1.27	1.82	C

S.N.: serial number of the analytes; M.P.: mobile phase: A: *n*-hexane/isopropanol (90/10); B: *n*-hexane/ethanol (90/10); C: *n*-hexane/ethanol/methanol (90/5/5). "+", "–", "R", "S", "4R,1S", "4S,1R" and "2R,3S)" at the superscript of k_1 refer to the optical rotation or configuration of the first-eluted enantiomer. Flow rate: 1 mL/min. Dectetion temperature: 25 °C.

4. Enantioseparation Recovery of CSP 5

Table S3. Enantioseparation of **CSP 5** under conditions I and V.

S.N.	Condition I			Condition V			M.P.
	k_1	α	R_s	k_1	α	R_s	
1	0.44	1.00	0.00	0.39	1.00	0.00	A
	0.34	1.37	1.54	0.31	1.28	1.40	B
	0.40	1.23	1.37	0.38	1.23	1.32	C
2	0.92	1.00	0.00	0.84	1.00	0.00	A
	0.62	1.00	0.00	0.57	1.00	0.00	B
	0.67	1.00	0.00	0.64	1.00	0.00	C
3	3.38	1.31	3.61	3.03	1.33	3.61	A
	2.26	1.18	2.08	2.06	1.22	2.25	B
	2.02	1.12	1.42	1.93	1.14	1.42	C
4	1.96	1.29	3.65	1.76	1.26	3.99	A
	1.26	1.27	3.01	1.16	1.29	2.99	B
	1.18	1.15	1.52	1.13	1.16	1.72	C
5	7.42	1.20	2.80	6.79	1.20	2.78	A
	3.57	1.16	2.25	3.29	1.17	2.25	B
	2.71	1.10	1.35	2.58	1.11	1.49	C
6	3.13	1.00	0.00	2.96	1.00	0.00	A
	2.19	1.00	0.00	2.13	1.00	0.00	B
	1.75	1.00	0.00	1.86	1.00	0.00	C
7	0.41	1.00	0.00	0.37	1.00	0.00	A
	0.47	1.00	0.00	0.42	1.00	0.00	B
	0.50	1.00	0.00	0.53	1.00	0.00	C
8	3.70	1.15	0.51	3.62	1.14	0.54	A
	2.57	1.00	0.00	2.44	1.00	0.00	B
	2.10	1.00	0.00	2.24	1.00	0.00	C
9	1.71	1.00	0.00	1.68	1.00	0.00	A
	0.91	1.00	0.00	0.95	1.00	0.00	B
	0.89	1.00	0.00	0.93	1.00	0.00	C
10	3.73	1.46	2.28	3.21	1.47	1.56	A
	1.38	1.24	2.20	1.37	1.20	2.14	B
	1.26	1.00	0.00	1.25	1.00	0.00	C
11	6.10	1.00	0.00	5.36	1.00	0.00	A
	2.63	1.14	1.53	2.30	1.16	1.60	B
	2.02	1.16	1.57	1.92	1.18	1.80	C
12	3.87	1.71	3.97	2.74	1.89	2.64	A
	1.92	2.16	8.46	1.79	2.20	8.71	B
	1.45	1.61	5.09	1.51	1.58	4.95	C

Table S3. *Cont.*

S.N.	Condition I			Condition V			M.P.
	k_1	α	R_s	k_1	α	R_s	
13	1.56	1.44	3.37	1.36	1.48	3.54	A
	0.93	1.98	9.80	0.87	2.03	8.52	B
	0.88	2.36	6.92	0.91	2.37	8.47	C
14	5.37	1.43	3.09	4.66	1.43	3.27	A
	2.42	1.28	1.23	2.38	1.24	2.07	B
	1.91	1.00	0.00	1.91	1.00	0.00	C
15	7.82	3.58	7.45	7.09	3.40	9.48	A
	1.87	2.48	10.65	1.81	2.49	9.06	B
	1.51	1.23	1.74	1.62	1.28	2.92	C
16	6.24	2.89	6.62	5.52	2.69	5.22	A
	1.44	2.08	6.33	1.39	2.06	6.75	B
	1.08	1.31	2.01	1.14	1.27	2.47	C
17	4.88	1.34	2.63	4.54	1.26	1.83	A
	1.65	1.14	2.09	1.66	1.09	1.16	B
	1.36	1.00	0.00	1.39	1.00	0.00	C
18	16.57	1.15	1.65	14.44	1.13	1.53	A
	11.07	1.10	1.32	9.56	1.11	1.28	B
	8.05	1.14	1.66	7.45	1.17	2.08	C
19	Retention time > 120 min			Retention time > 120 min			A
	10.90	1.48	3.87	10.19	1.45	3.72	B
	7.00	1.17	2.18	6.66	1.13	1.41	C
20	Retention time > 120 min			Retention time > 120 min			A
	3.85	5.83	19.50	3.54	5.76	19.07	B
	2.44	3.93	16.41	2.34	3.86	15.94	C

S.N.: serial number of the chiral compounds. M.P.: mobile phase: A: *n*-hexane/isopropanol (90/10); B: *n*-hexane/ethanol (90/10); C: *n*-hexane/ethanol/methanol (90/5/5). Flow rate: 1 mL/min; dectetion temperature: 25 °C. The conditions of CSP 5: I: after **CSP 5** had been initially tested for its enantioseparation capability in *n*-hexane/isopropanol (90/10), *n*-hexane/ethanol 90/10, *n*-hexane/ethanol/methanol (90/5/5) and then placed aside for one month; V: after **CSP 5** had been analyzed in condition IV (see Table 2) and then was placed aside for two months.
